# Markers of adiposity in HIV/AIDS patients: Agreement between waist circumference, waist-to-hip ratio, waist-to-height ratio and body mass index

**DOI:** 10.1371/journal.pone.0194653

**Published:** 2018-03-22

**Authors:** Christian Akem Dimala, Roland Cheofor Ngu, Benjamin Momo Kadia, Frank-Leonel Tianyi, Simeon Pierre Choukem

**Affiliations:** 1 Faculty of Epidemiology and Population Health, London School of Hygiene and Tropical Medicine, London, United Kingdom; 2 Orthopaedics Department, Southend University Hospital, Essex, United Kingdom; 3 Health and Human Development (2HD) Research Network, Douala, Cameroon; 4 Nuffield Department of Medicine, University of Oxford, Oxford, United Kingdom; 5 Medical Doctors (M.D) Research Group, Douala, Cameroon; 6 Foumbot District Hospital, Foumbot, Cameroon; 7 Grace Community Health and Development Association, Kumba, Cameroon; 8 Mayo-Darle District Hospital, Mayo-Darle, Cameroon; 9 Diabetes and Endocrinology Unit, Department of Internal Medicine, Douala General Hospital, Douala, Cameroon; 10 Department of Internal Medicine and Paediatrics, Faculty of Health Sciences, University of Buea, Buea, Cameroon; University of Alabama at Birmingham, UNITED STATES

## Abstract

**Background:**

Waist circumference (WC), waist-to-hip ratio (WHR) and waist-to-height ratio (WHtR) are all independent predictors of cardio-metabolic risk and therefore important in HIV/AIDS patients on antiretroviral therapy at risk of increased visceral adiposity. This study aimed to assess the extent of agreement between these parameters and the body mass index (BMI), as anthropometric parameters and in classifying cardio-metabolic risk in HIV/AIDS patients.

**Methods:**

A secondary analysis of data from a cross-sectional study involving 200 HIV/AIDS patients was done. Anthropometric parameters were measured from participants using standard guidelines and central obesity defined according to recommended criteria. Increased cardio-metabolic risk was defined according to the standard cut-off values for all four parameters. Data were analyzed using STATA version 14.1.

**Results:**

The prevalence of WC-defined central obesity, WHR-defined central obesity and WHtR > 0.50 were 33.5%, 44.5% and 36.5%, respectively. The prevalence of BMI-defined overweight and obesity was 40.5%. After adjusting for gender and HAART status, there was a significant linear association and correlation between WC and BMI (regression equation: WC (cm) = 37.184 + 1.756 BMI (Kg/m^2^) + 0.825 Male + 1.002 HAART, (p < 0.001, r = 0.65)), and between WHtR and BMI (regression equation: WHtR = 0.223 + 0.011 BMI (Kg/m^2^)– 0.0153 Male + 0.003 HAART, (p < 0.001, r = 0.65)), but not between WHR and BMI (p = 0.097, r = 0.13). There was no agreement between the WC, WHtR and BMI, and minimal agreement between the WHR and BMI, in identifying patients with an increased cardio-metabolic risk.

**Conclusion:**

Despite the observed linear association and correlation between these anthropometric parameters, the routine use of WC, WHR and WHtR as better predictors of cardio-metabolic risk should be encouraged in these patients, due to their minimal agreement with BMI in identifying HIV/AIDS patients with increased cardio-metabolic risk. HAART status does not appear to significantly affect the association between these anthropometric parameters.

## Introduction

The measurement of waist circumference (WC) is recommended as a routine clinical parameter during the follow-up of human immuno-deficiency virus (HIV)-infected patients [1]. WC is a simple, non-invasive and inexpensive parameter to measure; however, this is not routinely assessed in most clinical settings [2,3]. WC is a key parameter used in defining central obesity [4], and it provides important information on alterations in patient central adiposity hence it is significantly associated to the other parameters of the metabolic syndrome [5]. Its place in the day-to-day follow-up of HIV/AIDS patients cannot be overemphasized.

The body mass index (BMI) is an anthropometric parameter more frequently measured in clinical settings due to its comparability across different populations and areas in addition to its non-invasiveness [6]. BMI permits classification of patients as being overweight or obese hence predicting their cardio-metabolic risk [6,7]. However, the use of BMI in ascertaining cardio-metabolic risk is not without drawbacks, since several other factors such as important muscle mass, age, and ethnicity are not accounted for [6,8]. A patient with a raised BMI because of a significant muscle mass may not necessarily have a high body fat mass which is a better predictor of cardio-metabolic risk. Likewise, patients with abdominal obesity as a result of intra-abdominal visceral adiposity do not always present with an elevated BMI [9]. The fixed BMI categories used in predicting cardio-metabolic risk do not take into consideration ethnicity and age, on which overall mortality depends [10]. Nevertheless, BMI has been continuously used as a substitute to WC based on the assumption that these two parameters have a positive linear relationship [11]. The Waist-to-hip ratio (WHR) which is an even more accurate measure of central adiposity and predictor of cardio-metabolic risk [12] has been recommended by the World Health Organization (WHO) in defining the metabolic syndrome [13]. Likewise the Waist-to-Height Ratio (WHtR) was reported to be a better predictor of cardio-metabolic risk than BMI in a review by Savva *et al* [14]. This anthropometric parameter has the additional merit of not being dependent on age, sex or ethnicity, since the standard cut-off value of 0.5 has been found to be indicative of an increased cardio-metabolic risk in both children and adults, men and women and people of different ethnicities [14,15]. The use of such accurate and independent predictors of cardio-metabolic risk is particularly relevant in HIV-infected patients on antiretroviral therapy which is known to induce corporal fat redistribution towards central and visceral deposition [16,17]. The WHO has highlighted the place of measures of central obesity as better predictors of cardio-metabolic risk compared to BMI, but has however, recommended their complementary use with BMI due to the resultant improved discriminatory capability [18].

In most clinical settings, the choice on which parameter to routinely measure depends on several prevailing conditions such as the availability of measuring instruments, health personnel and ease of measurement of these measurements. Given these extensive variations in the parameters preferentially measured across different clinical settings and the importance of lipodystrophic changes in HIV/AIDS patients, we aimed to assess the agreement of these measures, first as anthropometric parameters of routine clinical practice, and secondly in classifying cardio-metabolic risk in HIV/AIDS patients.

### Objectives

1. To determine if there is a linear association between WC, WHR, WHtR and BMI in HIV/AIDS patients.
2. To determine the predicted BMI values corresponding to the WC, WHR and WHtR cut-off values for central obesity.
3. To assess the agreement between WC, WHR, WHtR and BMI in identifying patients with an increased cardio-metabolic risk.

## Methods

### Study design and participants selection

This was a secondary analysis of data from a cross-sectional study involving HIV/AIDS patients who visited the HIV treatment center of the Limbe Regional Hospital in the South West Region of Cameroon, during the second quarter of 2013 [19]. This major referral center caters for more than 6500 HIV/AIDS patients. A total of two hundred participants were recruited in the primary study. The participants selection, study procedures, data sources and measurements are described in detail in this previous report [19].

### Participants and sampling

Randomly sampled participants among the patients attending routine follow-up visits were enrolled into the study if they were above 21 years of age and they consented to take part in the study.

### Study procedures and variables

Eligible participants who consented to participate were administered a standardized questionnaire through a face-to-face interview and physically examined to measure individual anthropometric parameters. Collected data consisted of participants’ socio-demographics, medical and social histories, blood pressure (BP), weight, height, waist and hip circumferences.

### Data sources and measurements

WC was measured to the nearest half centimeter using a measuring tape midway between the iliac crest and the lower rib margin [18]. Based on the African ethnicity of the study population, central obesity was defined as a WC > 94 cm in men and > 80 cm in women [18]. These cut-offs were considered as indicative of an increased cardio-metabolic risk [18]. The hip circumference, reported to the nearest half centimeter, was measured at the intertrochanteric level [18]. The WHR was derived using the formula: waist (cm)/hip (cm) circumferences and reported to 2 decimal places. The WHR-defined central obesity was defined as a WHR ˃ 0.90 in men and WHR ˃ 0.85 in women [18] and considered as indicative of an increased risk for cardio-metabolic disease [18]**.** Weight was measured to the nearest half kilogram using a scale (Brand—BRN 9311) with patients wearing minimal clothing that had little or no effect on their overall weight. Height was measured to the nearest half centimeter with a stadiometer. The WHtR was derived using the formula: waist circumference (cm)/height (cm) and reported to 2 decimal places. A WHtR above 0.50 in both males and females was considered to indicate an increased cardio-metabolic risk [20]. Body mass index, reported to 2 decimal places in Kg/m^2^, was derived using the conventional formula: weight (kg)/[height (m) X height (m)] and categorized as: Underweight (< 18.5 Kg/m^2^), normal (18.5–24.9 Kg/m^2^), overweight (25 to 29.9 Kg/m^2^) and obesity (≥30 Kg/m^2^) [21,22]. The recommended cut-off of BMI ≥25 Kg/m^2^ was used as indicative of an increased cardio-metabolic risk [21].

### Data management and data analysis

Data were entered into Epi-info version 7 and analyzed using STATA version 14.1 statistical software. For objective 1, respective linear regression models were built to determine if the associations between WC, WHR, WHtR and BMI are linear. Regression equations best describing the association between these parameters were derived. Multiple regression was used to derive regression equations while controlling for gender and HAART status. For objective 2, WC, WHR and WHtR cut-off values for central obesity or increased cardio-metabolic risk were used to predict the corresponding BMI values from the respective regression models. For objective 3, the Cohen’s kappa statistic [23] was used to evaluate the extent of agreement between WC, WHR, WHtR and BMI, in identifying participants with an increased cardio-metabolic risk. The agreement was interpreted for various kappa statistic values as: Values between 0–0.20 as indicating no agreement, values between 0.21–0.39 as minimal agreement, values between 0.40–0.59 as weak agreement, values between 0.60–0.79 as moderate agreement, values between 0.80–0.90 as strong agreement and values > 0.90 as almost perfect agreement [23]. Missing data were excluded from the analyses.

### Ethical considerations and reporting

Ethical approval was previously obtained from the Institutional Review Board of the University of Buea to conduct the initial study. Ethical approval was not required for this secondary data analysis. The ‘Strengthening the Reporting of Observational studies in Epidemiology’ (STROBE) guidelines were used for reporting this study (S1 Table).

## Results

### Socio-demographic and clinical characteristics of the study population

Two hundred participants were enrolled in the primary study. All clinical and socio-demographic characteristics of the study population are summarized on Table 1. Among all participants, 33.5% and 44.5% had WC-defined and WHR-defined central obesity respectively. Likewise, 36.5% had a WHtR above normal and 40.5% had BMI-defined overweight. Half of the participants were on highly active antiretroviral therapy (HAART) for a median duration of 60 months (IQR: 36–72 months). The prevalence of BMI-defined overweight and elevated WHtR were significantly higher in patients on HAART compared to HAART-naïve patients, while there was no significant difference in the prevalence of WC-defined and WHR-defined central obesity between the two groups (Table 2).

**Table 1:** Socio-demographic and clinical characteristics of all participants and according to gender.

Characteristic	Participants (n = 200)	Females (n = 140)	Males (n = 60)	P value
Age in years (mean ± SD)	39.1±9.4	38.4±9.8	40.7±8.3	0.123
Married, n (%)	89 (44.5%)	49 (35%)	40 (66.7%)	<0.001
Waist circumference in cm (mean ± SD)	80.3±7.9	80.2±7.9	80.7±7.7	0.660
WC-defined central obesity, prevalence (%)	33.5%	94.0%	6.0%	<0.001
Hip circumference in cm (mean ± SD)	94.0±8.0	94.2±8.3	93.6±7.3	0.635
WHR (mean ± SD)	0.86±0.06	0.85±0.06	0.86±0.07	0.277
WHR-defined central obesity, prevalence (%)	44.5%	52.9%	25.0%	<0.001
Weight in Kg (mean ± SD)	65.8±9.4	64.2±9.5	69.4±8.4	0.270
Height in meters (mean ± SD)	1.65±0.07	1.63±0.07	1.70±0.06	0.926
WHtR (mean ± SD)	0.49±0.05	0.49±0.05	0.48±0.05	0.024
Elevated WHtR (WHtR > 0.5, prevalence (%))	36.5%	40.7%	26.7%	0.059
BMI in Kg/m^2^ (mean ± SD)	24.1±2.9	24.2±3.0	24.0±2.5	0.709
BMI-defined overweight, prevalence (%)	40.5%	44.29%	31.67%	0.096
BMI categories				0.140
*Underweight*	2 (1.0%)	2 (1.4%)	0 (0.0%)	
*Normal*	117 (58.5%)	76 (54.3%)	41 (68.3%)	
*Overweight*	81 (40.5%)	62 (44.3%)	19 (31.7%)	
*Obesity*	0 (0.0%)	0 (0.0%)	0 (0.0%)	
Hypertension (prevalence, %)	28.5%	22.1%	43.3%	0.002
Time since diagnosis of HIV infection in months, median (IQR)	22 (1–68)	18 (1–62)	24 (1–72)	0.860
CD4 cell count in cells/µL, median (IQR) (n = 123)	271 (130–408)	270 (134–409)	274 (80–388)	0.646

BMI–Body Mass Index, CD4—Cluster of differentiation 4, IQR–Interquartile Range, SD–Standard deviation, WC–Waist circumference, WHR–Waist-to-hip ratio, WHtR–Waist-to-height ratio.

**Table 2:** Socio-demographic and clinical characteristics according to antiretroviral therapy status.

Characteristic	HAART (n = 100)	HAART-naïve (n = 100)	P value
Age in years (mean ± SD)	40.2±8.0	38.0±10.6	0.106
Married, n (%)	52 (52%)	37 (37%)	0.033
Waist circumference in cm (mean ± SD)	81.9±7.5	78.8±7.9	0.004
WC-defined central obesity (prevalence, %)	40%	27%	0.051
Hip circumference in cm (mean ± SD)	95.3±6.6	92.7±9.1	0.021
WHR (mean ± SD)	0.86±0.07	0.85±0.06	0.333
WHR-defined central obesity (prevalence, %)	46%	43%	0.669
Weight in Kg (mean ± SD)	67.7±8.5	63.8±10.0	0.004
Height in meters (mean ± SD)	1.65±0.1	1.64±0.1	0.365
WHtR (mean ± SD)	0.50±0.05	0.48±0.05	0.016
Elevated WHtR (WHtR > 0.5, (prevalence, %))	44%	29%	0.028
BMI in Kg/m^2^ (mean ± SD)	24.8±2.8	23.5±2.8	0.002
BMI-defined overweight (prevalence, %)	50%	31%	0.006
BMI categories			0.023
*Underweight*	1%	1%	
*Normal*	49%	68%	
*Overweight*	50%	31%	
*Obesity*	0%	0%	
Hypertension (prevalence, %)	38 (38%)	19 (19%)	0.003
Time since diagnosis of HIV infection in months, median (IQR)	68 (42–84)	1 (1–1)	<0.001
CD4 cell count in cells/μL, median (IQR), (n = 123)	476 (361–619)	178 (70–273)	<0.001

BMI–Body Mass Index, CD4—Cluster of differentiation 4, IQR–Interquartile Range, SD–Standard deviation, WC–Waist circumference, WHR–Waist-to-hip ratio, WHtR–Waist-to-height ratio.

### Association between WC, WHR, WHtR and BMI

There was a strong linear association and correlation between WC and BMI among the study participants (p < 0.001, r = 0.65) and according to gender and HAART status from the respective linear regression models (Table 3). On multiple regression controlling for gender and HAART status, there was still a strong linear association between WC and BMI, with the regression equation being:

WC (cm) = 37.184 + 1.756 BMI (Kg/m^2^) + 0.825 Male + 1.002 HAART, (p < 0.001, R^2^ = 42.9%).

**Table 3:** Linear regression model for waist circumference, waist-to-hip ratio and body mass index for the study population, according to gender and HAART status.

Parameters	Regression Coefficient	Intercept	P value	R-square	Correlation Coefficient
**Waist Circumference**					
Overall	1.790	37.103	<0.001	0.42	0.65
Females	1.742	38.029	<0.001	0.44	0.66
Males	1.978	33.168	<0.001	0.40	0.63
HAART	1.627	41.617	<0.001	0.36	0.60
HAART-naïve	1.876	34.602	<0.001	0.44	0.66
**Waist-to-Hip Ratio**					
Overall	0.003	0.787	0.072	0.02	0.13
Females	0.003	0.787	0.121	0.02	0.13
Males	0.004	0.777	0.990	0.02	0.13
HAART	0.007	0.677	0.001	0.10	0.32
HAART-naïve	-0.002	0.901	0.365	0.00	-0.09
**Waist-to-Height Ratio**					
Overall	0.011	0.215	<0.001	0.42	0.65
Females	0.011	0.229	<0.001	0.43	0.66
Males	0.012	0.180	<0.001	0.41	0.65
HAART	0.011	0.213	<0.001	0.41	0.64
HAART-naïve	0.011	0.223	<0.001	0.41	0.64

HAART–Highly Active Antiretroviral Therapy

No linear association and correlation was observed between the WHR and BMI in the entire study population (p = 0.072, r = 0.13). No correlation was noted between WHR and BMI among HAART-naïve patients (r = -0.09) (Table 3). The multiple regression equation for WHR and BMI while controlling for gender and HAART status is:

WHR = 0.785 + 0.003 BMI (Kg/m^2^) + 0.011 Male + 0.005 HAART, (p = 0.097, R^2^ = 2.5%).

There was also a strong linear association and correlation between the WHtR and BMI among the entire study participants (p < 0.001, r = 0.65) and according to gender and HAART status (Table 3). This linear association was still present even after adjusting for gender and HAART status on multiple regression:

WHtR = 0.223 + 0.011 BMI (Kg/m^2^)– 0.0153 Male + 0.003 HAART, (p < 0.001, R^2^ = 44.4%).

Figs 1–9 present scatter diagrams for the relationship between WC, WHR, WHtR and BMI in the general study population, and according to gender and HAART status respectively.

**Fig 1:**
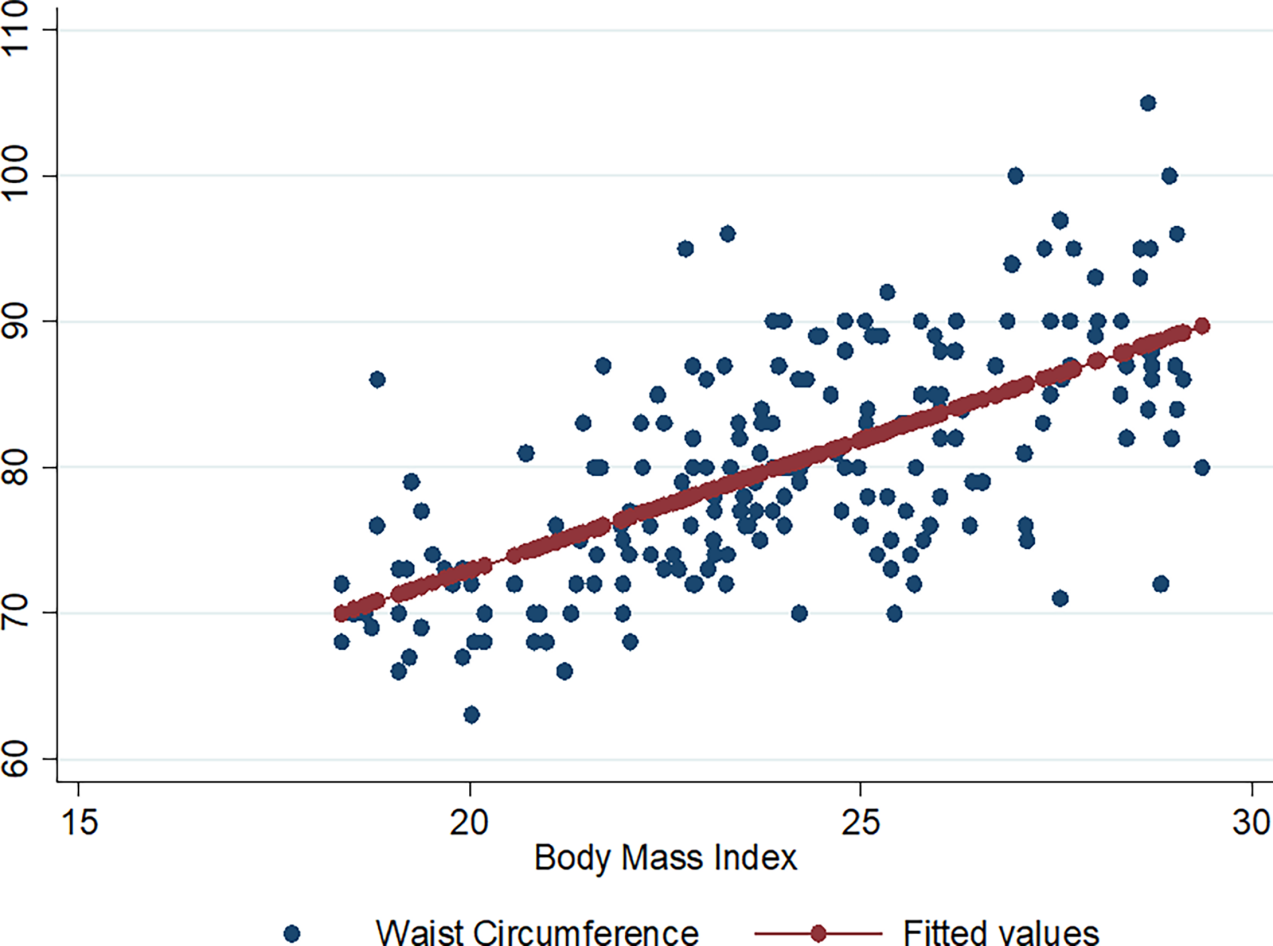
Linear regression model of the association between WC and BMI in all study participants.

**Fig 2:**
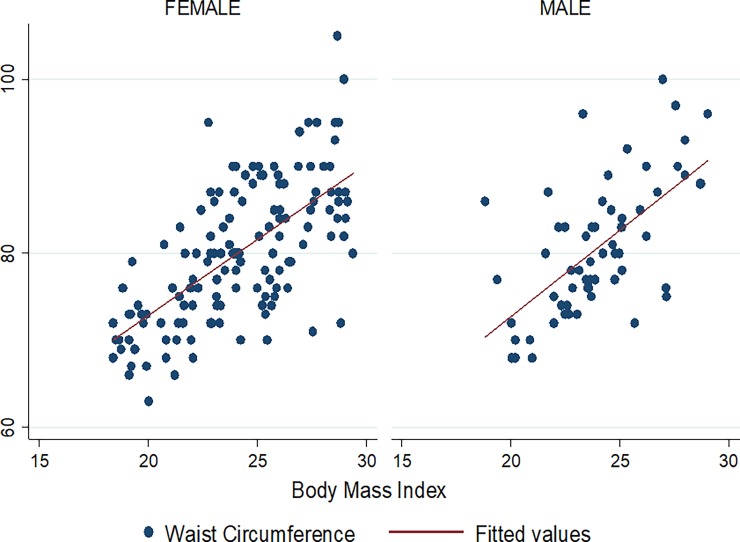
Linear regression model of the association between WC and BMI in females and males.

**Fig 3:**
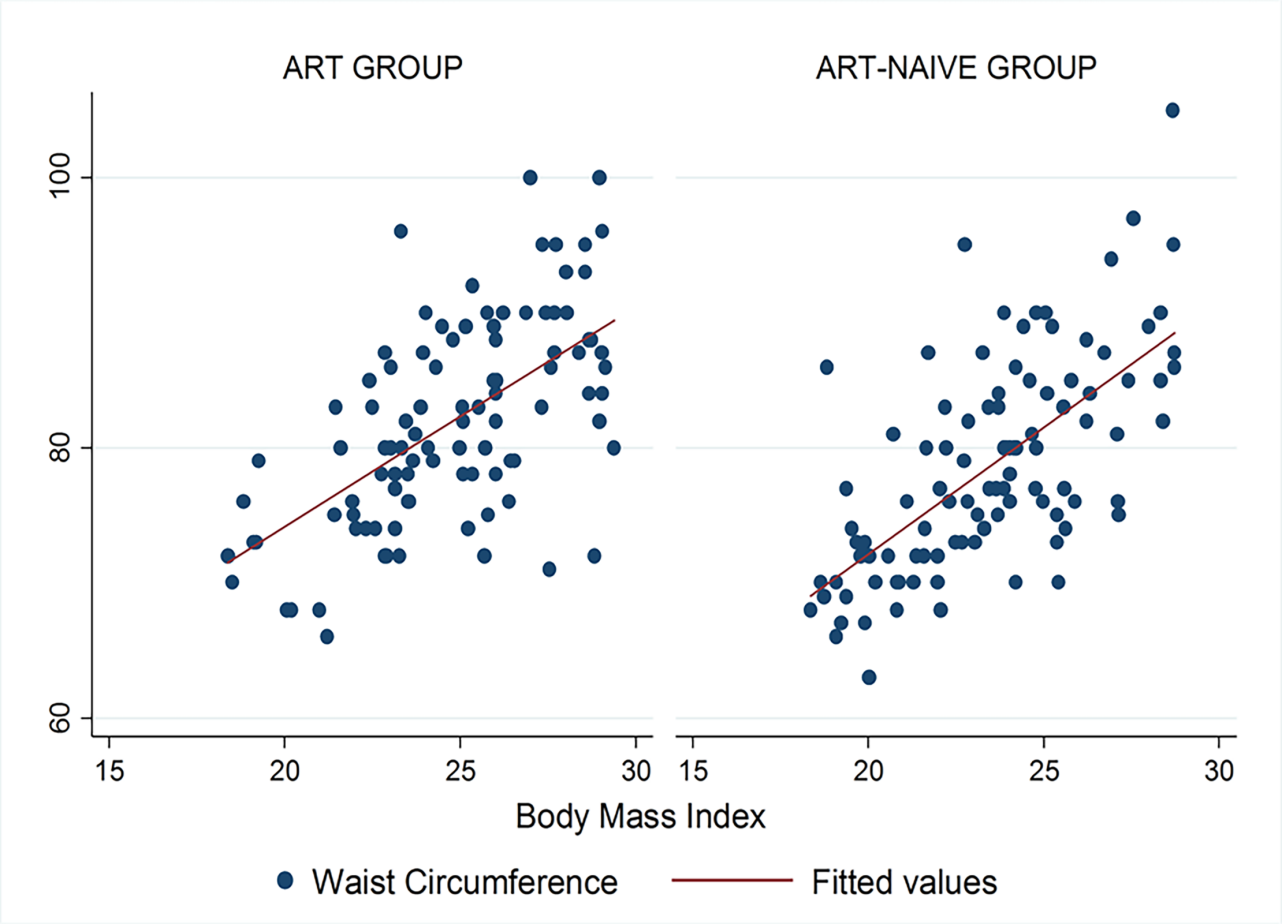
Linear regression model of the association between WC and BMI according to HAART status.

**Fig 4:**
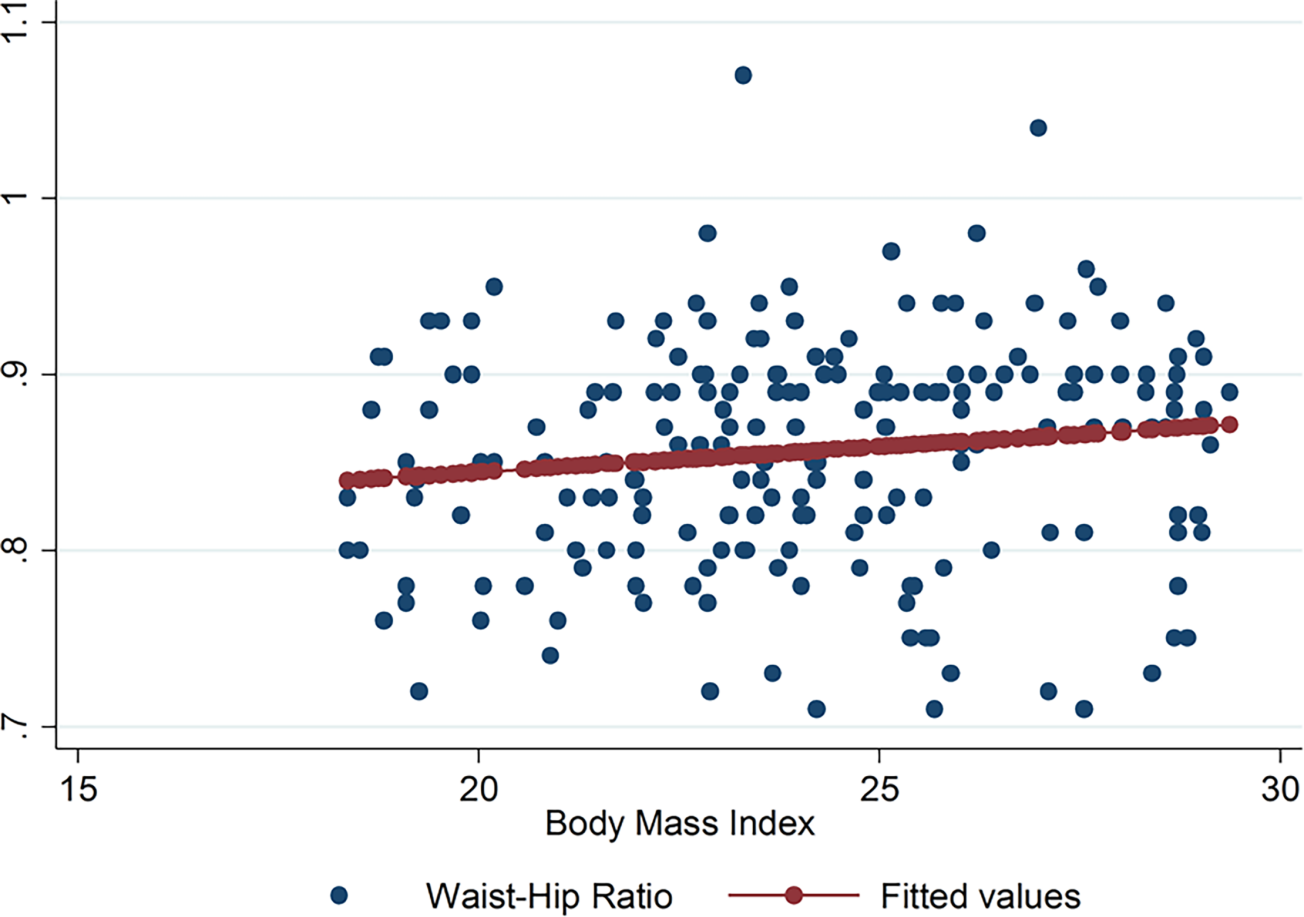
Linear regression model of the association between WHR and BMI in all study participants.

**Fig 5:**
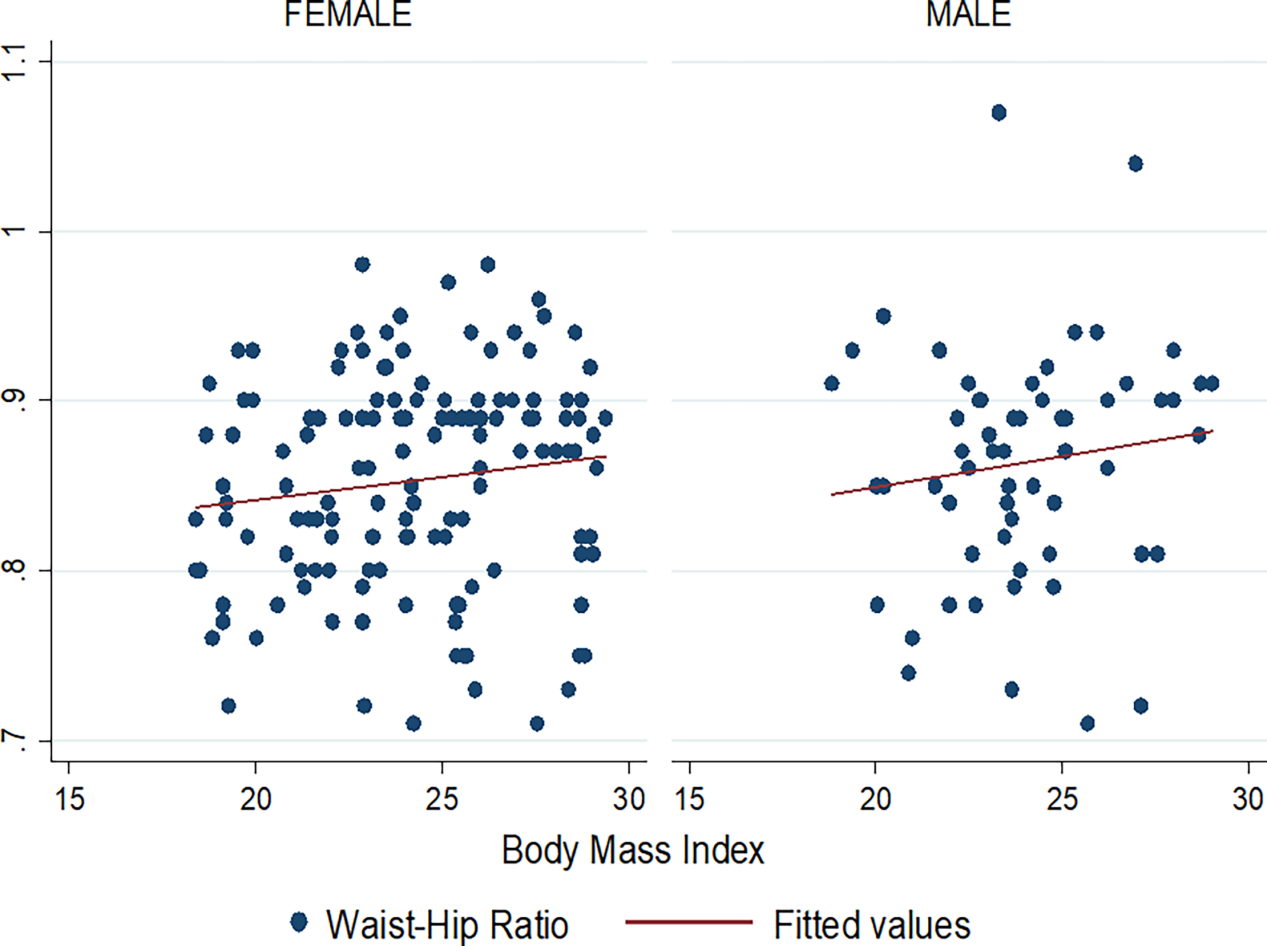
Linear regression model of the association between WHR and BMI in females and males.

**Fig 6:**
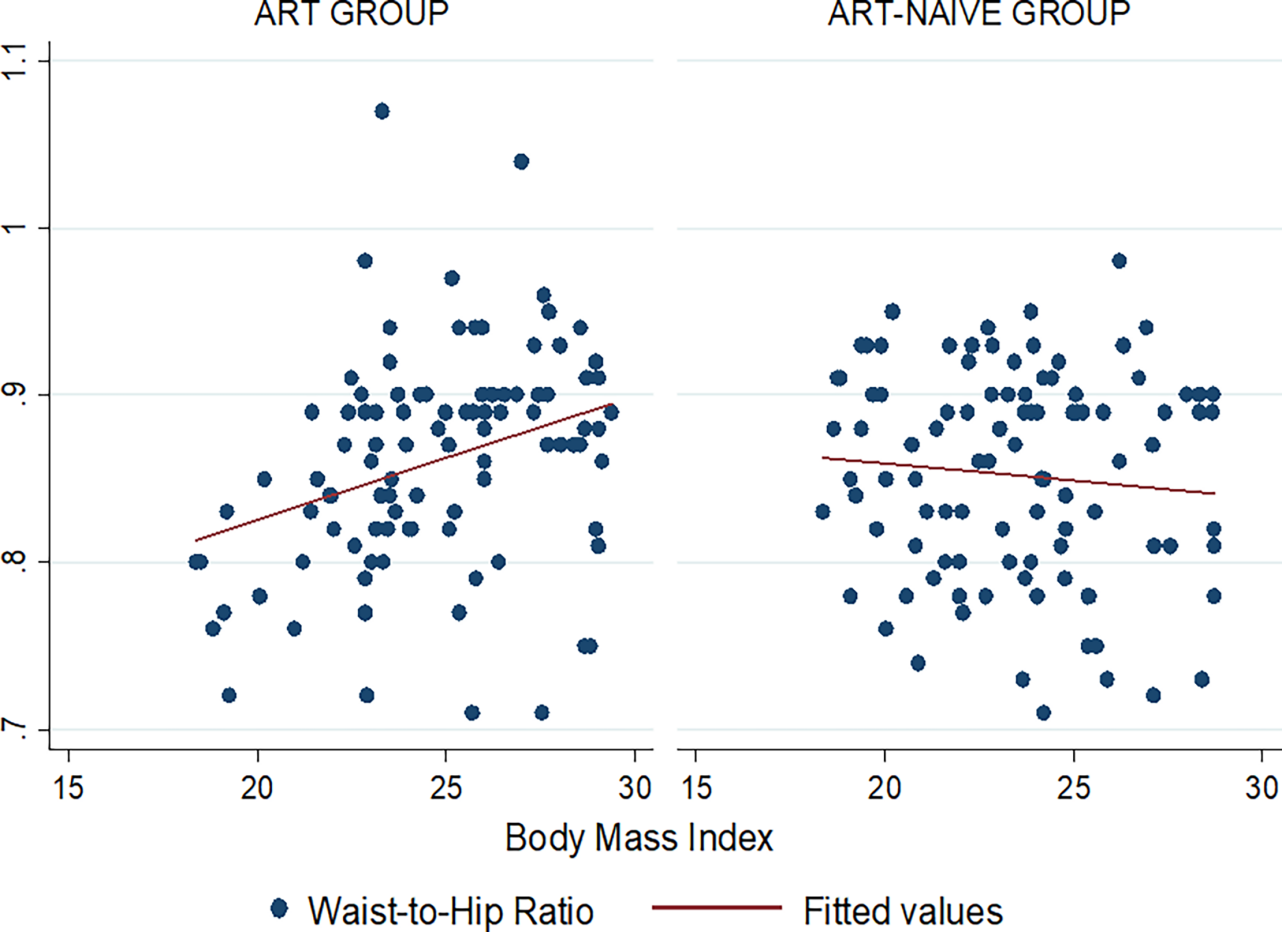
Linear regression model of the association between WHR and BMI according to HAART status.

**Fig 7:**
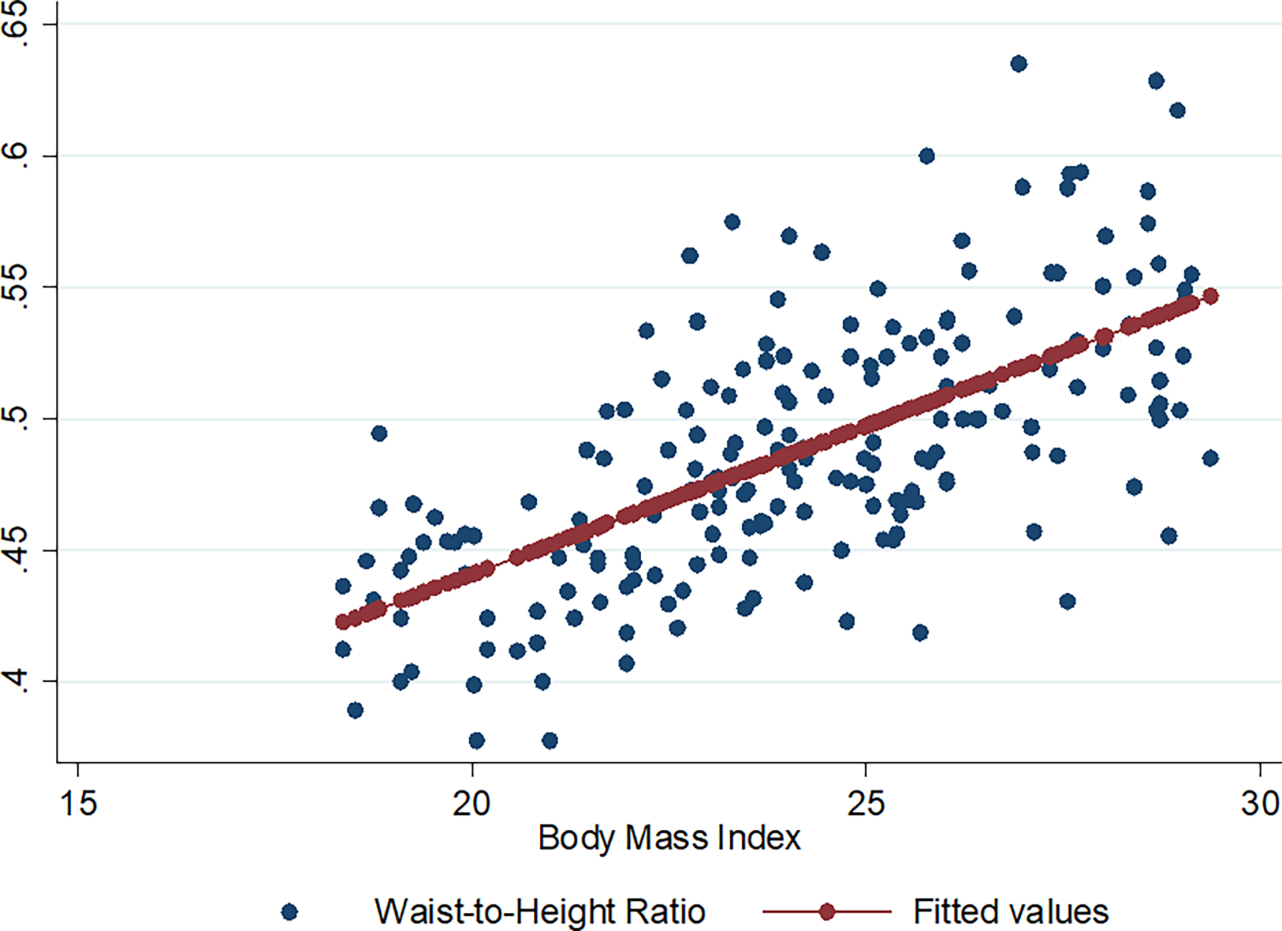
Linear regression model of the association between WHtR and BMI in all study participants.

**Fig 8:**
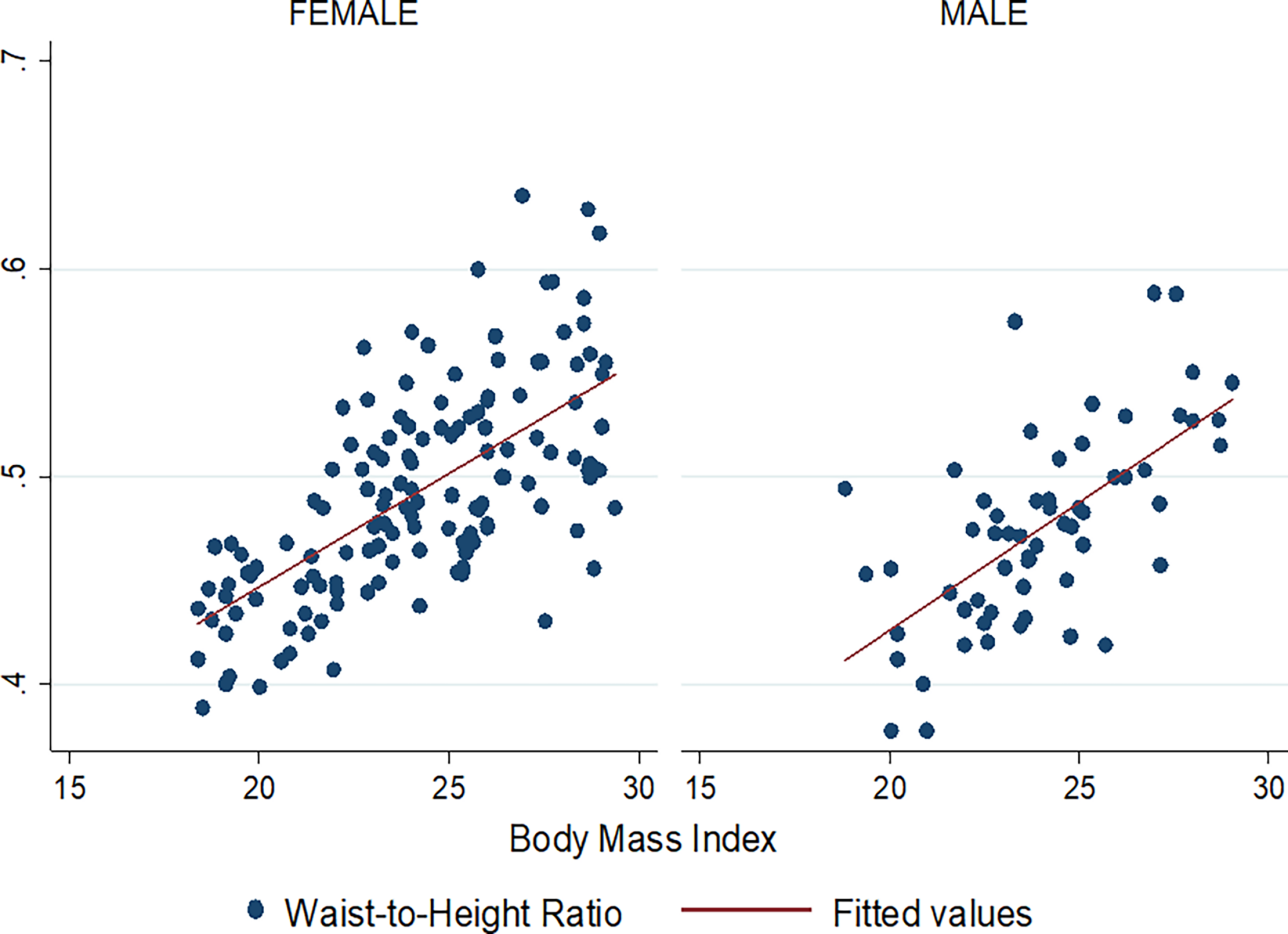
Linear regression model of the association between WHtR and BMI in females and males.

**Fig 9:**
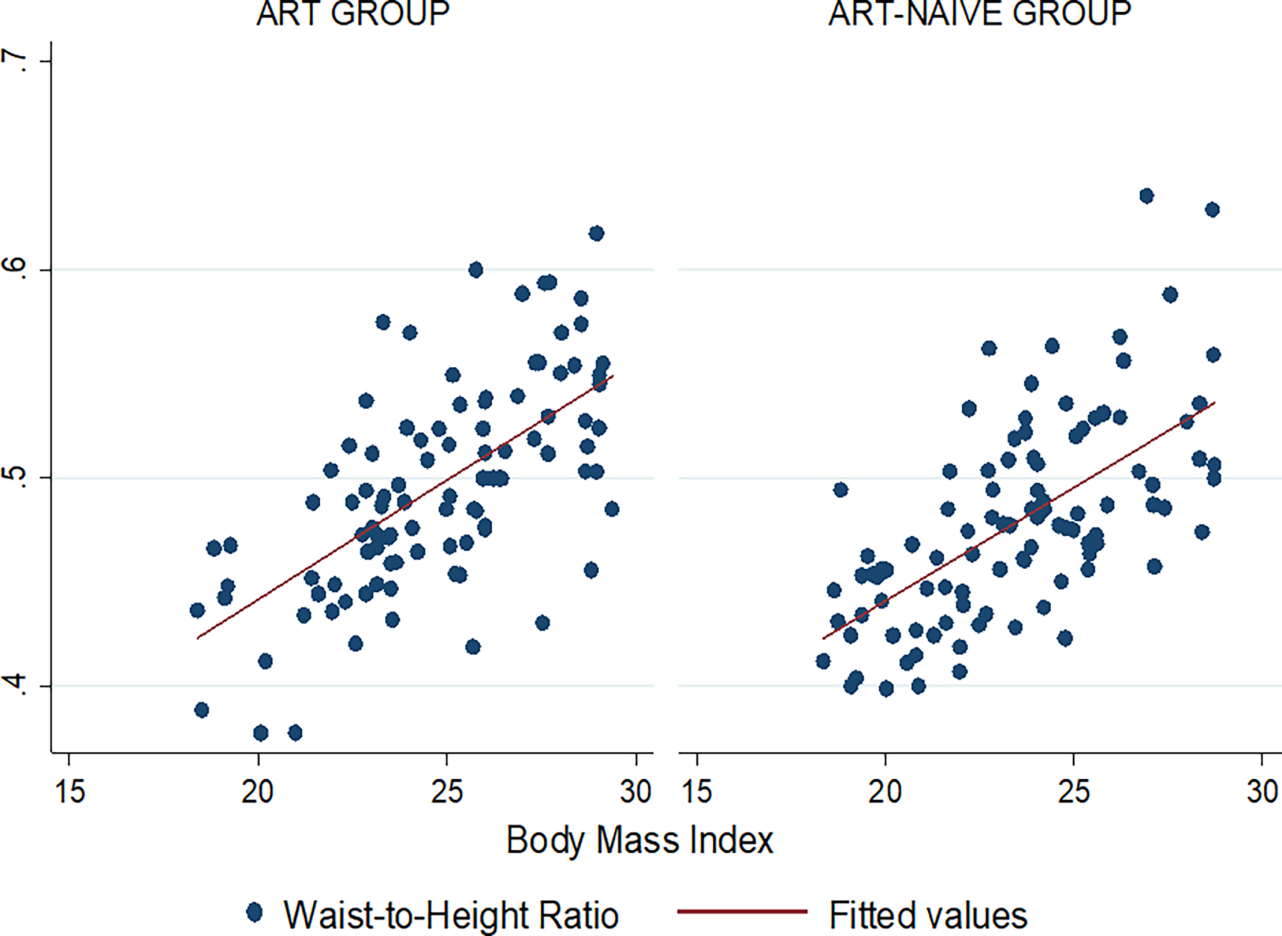
Linear regression model of the association between WHtR and BMI according to HAART status.

### Predicted BMI values for WC, WHR and WHtR cut-off values for increased cardio-metabolic risk

The predicted BMI values corresponding to the WC cut-off values for central obesity (80 cm in women and 94 cm in men) were 24.1 Kg/m^2^ and 30.8 Kg/m^2^ in females and males respectively. Likewise, WHR values of 0.85 and 0.90 used as lower limits to define central obesity corresponded to BMI values of 23.1 Kg/m^2^ and 34.1 Kg/m^2^ in females and males respectively. WHtR > 0.50 in both females and males corresponded to a predicted BMI value of 24.9 Kg/m^2^ and 26.0 Kg/m^2^ in females and males respectively.

### Agreement between WC, WHR, WHtR and BMI in assessing cardio-metabolic risk

There was no agreement between the WC, WHtR and BMI in predicting an increased cardio-metabolic risk among participants (Kappa Statistic = 0.16 and p < 0.001 for both) (Table 4). Likewise the WHR and BMI had minimal agreement in identifying patients with an increased risk (Kappa Statistic = 0.20, p = 0.002) (Table 4). In contrast to the similar agreements observed between the WC, WHtR and BMI among HAART and HAART-naïve patients, the agreement between WHR and BMI was significantly higher in the HAART group compared to the HAART-naïve group (Table 5).

**Table 4:** Agreement between WC, WHR, WHtR and BMI in identifying patients with an increased cardio-metabolic risk.

Parameters	Body Mass Index			Agreement	Kappa	P value
	Increased risk					
	Yes	No	Total			
**Waist circumference Increased risk**				49.5%	0.16	<0.001
Yes	47	20	67			
No	34	99	133			
Total	81	119	200			
**Waist-to-Hip Ratio Increased risk**				61.0%	0.20	0.002
Yes	46	43	89			
No	35	76	111			
Total	81	119	200			
**Waist-to-Height Ratio Increased risk**				47.5%	0.16	<0.001
Yes	49	24	89			
No	32	95	111			
Total	81	119	200			

**Table 5:** Agreement between WC, WHR, WHtR and BMI in identifying patients with an increased cardio-metabolic risk according to HAART status.

Parameter	Agreement	Kappa Statistic	P value
**Waist Circumference**			
HAART	60%	0.20	0.001
HAART-naive	71%	0.17	0.018
**Waist-to-Hip Ratio**			
HAART	68%	0.36	<0.001
HAART-naïve	54%	0.03	0.385
**Waist-to-Height Ratio**			
HAART	39%	0.15	<0.001
HAART-naïve	56%	0.14	<0.001

## Discussion

In this study, we sought to assess the extent of agreement between WC, WHR, WHtR and BMI, initially as clinical parameters and also as surrogates of cardio-metabolic risk. We found a strong linear association and correlation between the WC, WHtR and BMI even after adjusting for gender and HAART status, but not between the WHR and BMI. However, the WC, WHtR and WHR had no and minimal agreements respectively, with the BMI in identifying HIV/AIDS patients with an increased cardio-metabolic risk. The WC and WHR cut-off values for defining central obesity did not match the reference BMI cut-off value of 30 Kg/m^2^ used for defining obesity, while the WHtR cut-off value corresponded closely to the BMI cut-off used for defining overweight.

There was a significant linear association and positive correlation between WC and BMI both as continuous variables in HIV/AIDS patients. This indicates that there is a proportional rise in the WC as the BMI increases. A much stronger correlation between WC and BMI was reported in the study by Pereira *et al* (r = 0.90, p < 0.001) in a population of adolescent females [24] and a slightly stronger correlation (r = 0.78) was noted in the study by Gierach *et al* [25], possibly due to a study population constituted entirely of participants with metabolic syndrome. Among HIV-infected patients, De Socio *et al* also had a slightly stronger correlation between both anthropometric parameters in both men (r = 0.73) and women (r = 0.74) [9]. This study, however, had a higher proportion of participants on HAART (69%) compared to our study (50%), supporting the evidence of the probable role of HAART in increasing central adiposity, in addition to other factors such as dietary intake known to considerably affect central obesity [26]. It is, however, worth noting that the WC and BMI correlation was stronger in the HAART-naïve group compared to the HAART group in this study.

Despite these minor differences among these studies, they all recorded a correlation of greater magnitude between WC and BMI among females compared to males participants, possibly due to the greater adipose tissue deposition and overall body fat in women compared to men [27]. On the other hand, no linear association was observed between WHR and BMI in our study participants, and as earlier reported by Pereira *et al* (r = 0.28, p < 0.01) [24]. This suggests that BMI increases in HIV-infected patients is not always accompanied by similar increases in the WHR. This is potentially due to the unnoticed alterations in body fat composition occurring in the peripheral compartment concurrent with those occurring centrally, as earlier emphasized by Brown *et al* [28]. A negative correlation between WHR and BMI was rather observed in HAART-naïve patients in our study. A linear association and positive correlation was also observed between the WHtR and BMI in the entire study population, though not as strong as that recorded by Pereira *et al* in an entirely female cohort (r = 0.89, p < 0.01) [24].

Significantly higher prevalence of both WC-defined central obesity and WHR-defined central obesity were recorded in females compared to males in our study, indicating a greater cardio-metabolic risk in female patients. In as much as health policies should emphasize on the systematic monitoring of WC in HIV/AIDS patients, a greater emphasis should be laid on females, who not only have a greater risk of contracting HIV infection, but are also more likely to develop cardio-metabolic diseases.

From the regression equations, the predicted BMI values in females corresponding to the WC, WHR and WHtR cut-off values for central obesity and increased cardio-metabolic risk were lower than the 30 Kg/m^2^ used to define obesity. This suggests that an increased cardio-metabolic risk should be expected in female HIV/AIDS patients at BMI values lower than the usual 30 Kg/m^2^ obesity cut-off and appropriate measures taken.

Using the standard cut-off values of all four anthropometric parameters, WHR and WC were the most and least sensitive in detecting increased cardio-metabolic risk respectively. This supports the findings of the review by de Koning *et al* in which WHR had a stronger association to cardiovascular disease compared to WC [29]. Even though WC is helpful in detecting abdominal obesity, its lower sensitivity in detecting cardio-metabolic risk compared to the WHR is due to its inability to differentiate between visceral fat deposition (independent predictor of cardio-metabolic risk) and abdominal subcutaneous fat deposition [8]. A large prospective cohort study also confirmed WHR as the most accurate predictor of cardiovascular events when compared to WC and BMI. [30]. Conversely, BMI has been considered as the least accurate predictor of cardiovascular events due to its inability to differentiate between fat and free-fat changes in overall body weight [8,30].

Despite the linear associations existing between these anthropometric parameters, we noted low kappa statistics values suggestive of minimal agreement between them in identifying participants with increased cardio-metabolic risk. These low kappa statistic values should, however, be interpreted with caution since this statistical parameter is grossly affected by the prevalence of the characteristic being investigated and it is less reliable for rare outcomes [31]. As such, low kappa values are not always suggestive of poor agreement rates for rare outcomes [31]. This is supported by the high unadjusted agreement percentages observed among the anthropometric parameters in our study (Table 5). Better agreements were noted in HAART patients compared to HAART-naïve patients, even though HAART status did not appear to significantly affect the association and correlation between these anthropometric parameters. Given the morphologic changes in HIV/AIDS patients and the associated cardio-metabolic risk, the routine use of WC, WHR, and WHtR in assessing cardio-metabolic risk in HIV/AIDS patients should be encouraged, and appropriate considerations taken in settings where BMI is still routinely used as the single anthropometric parameter.

This study is particularly relevant as it is the first study to compare these various anthropometric parameters of routine clinical practice in HIV/AIDS patients in Cameroon. Nevertheless, the interpretation of these findings should make allowance for some limitations of the study such as the cross-sectional design of the study making causal inferences inappropriate, and the use of general cut-off values of the measured anthropometric parameters in a study population of black ethnicity. Likewise, this may compromise the generalizability of these results to populations of other ethnicities. Despite these limitations, the findings of this study provide a relevant baseline from which other studies can build on. The routine measurement of all among HIVAIDS patients would certainly be complementary in identifying those with increased cardio-metabolic risk and allowing them deserved care.

## Conclusion

Given the morphologic changes that occur in HIV/AIDS, the use of better predictors of cardio-metabolic risk (WC, WHR and WHtR) should be encouraged despite the observed significant linear association and correlation between them and the BMI. This is because of their minimal agreement with BMI in identifying HIV/AIDS patients with increased cardio-metabolic risk, suggesting they potentially identify different at-risk populations. Nevertheless, settings still relying exclusively on BMI to assess cardio-metabolic risk should take into consideration the proposed BMI cut-off values. HAART does not appear to significantly affect the association between these anthropometric parameters.

## Supporting information

S1 Table STROBE checklist. (DOC)

S1 Data Dataset on the extent of agreement of waist circumference, waist-hip ratio and body mass index as markers of adiposity in HIV/AIDS patients. (DTA)

## Abbreviations

AIDS: Acquired immunodeficiency Virus
BMI: Body Mass Index
HAART: Highly Active Anti-retroviral Therapy
HIV: Human Immunodeficiency Virus
STROBE: Strengthening the Reporting of Observational studies in Epidemiology
WC: Waist Circumference
WHR: Waist-to-Hip Ratio
WHtR: Waist-to-Height Ratio
